# Case Report: Dual resistance to dasatinib/olverembatinib in accelerated-phase cml: identification of a novel *SPECC1L*-inserted e8a2 *BCR::ABL1* transcript and *ABL1* V379I mutation

**DOI:** 10.3389/fonc.2025.1711888

**Published:** 2025-10-24

**Authors:** Jingjing Fu, Yangming Tang, Xueqin Ruan, Runfa Wang, Tong Chen, Li Jiang, Yanli He, Zhihong Xu, Balian Wang, Haiqin Zhang, Jing Zhou, Mei Lan, Hongrui Li

**Affiliations:** ^1^ The Department of Cytogenetics, Kindstar Globalgene Technology, Inc, Wuhan, China; ^2^ The Department of Hematology, Guangxi Academy of Medical Sciences & The People’s Hospital of Guangxi Zhuang Autonomous Region, Nanning, China; ^3^ Center for Stem Cell Research and Application, Union Hospital, Tongji Medical College, Huazhong University of Science and Technology, Wuhan, China

**Keywords:** *BCR::ABL1*, e8a2, *SPECC1L*, chronic myeloid leukemia (CML), accelerated phase, V379I mutation

## Abstract

In chronic myeloid leukemia (CML), less than 2% of cases express atypical or rare *BCR::ABL1* transcripts. The e8a2 *BCR::ABL1* fusion transcript, a rare variant, has been reported in only 20 cases to date, primarily in case reports or case series. The direct junction between *BCR* exon 8 and *ABL1* exon 2 generates a premature stop codon at position 7 after the fusion, while the insertion of certain sequences can result in the formation of an in-frame e8a2 transcript. To date, the insertion of *SPECC1L* gene sequences into e8a2 *BCR::ABL1* fusion transcripts has been reported in two CML cases, and the V379I mutation (in *ABL1*) has been identified in two additional CML cases. We describe a case of accelerated-phase CML involving three key molecular abnormalities: the insertion of a 154 bp *SPECC1L* exon 4 sequence into the e8a2 *BCR::ABL1* fusion transcript, a concomitant *ABL1* V379I mutation, and deletions near the t(9;22) breakpoint on derivative chromosome 9 (der(9)). The patient’s clinical manifestations, cytogenetic features, and molecular genetic characteristics were summarized and discussed. Despite sequential therapy with full-dose dasatinib for 10 months and the third-generation tyrosine-kinase inhibitor (TKI) olverembatinib for 7 months, the patient experienced progressive disease. She ultimately achieved Major Molecular Response (MMR) after haploidentical hematopoietic stem-cell transplantation (haplo-HSCT). This case highlights the importance of comprehensive molecular profiling at diagnosis and the need to develop alternative therapeutic strategies for rare *BCR::ABL1* variants.

## Introduction

CML is a hematopoietic stem cell malignancy characterized by a reciprocal translocation between chromosomes 9 and 22 [t(9;22)(q34;q11.2)], which results in the formation of the Philadelphia (Ph) chromosome and the *BCR::ABL1* fusion gene (1). This fusion gene encodes a protein with constitutively activated tyrosine kinase activity (2). Most CML cases harbor a *BCR::ABL1* fusion gene encoding the 210-kDa BCR::ABL1 protein, which arises from e13a2 (b2a2) and/or e14a2 (b3a2) junctions depending on the breakpoint location within the *BCR* gene (3). However, in less than 2% of cases (4), the breakpoint occurs outside the major *BCR* (M-*BCR*) region or is fused with an exon other than *ABL1* exon 2, leading to the generation of atypical transcripts such as e1a2, e19a2, e13a3, e14a3, e1a3, e6a2, e8a2, and others (2, 3). The translation of each different transcript results in distinct protein tyrosine kinases, which may potentially affect the biological characteristics of the disease and the response to treatment (2).

Although e8a2 *BCR::ABL1* transcripts have been reported previously (5, 6), we describe a rare case of CML in which the e8a2 transcript contains an in-frame 154 bp insertion derived from *SPECC1L* exon 4 between *BCR* exon 8 and *ABL1* exon 2. Additionally, an acquired *ABL1* V379I mutation in exon 7 and deletions near the t(9;22) breakpoint on der(9) were identified. The patient became resistant to imatinib, and subsequent therapy with dasatinib and olverembatinib failed to achieve the expected outcomes. She therefore underwent haplo-HSCT in July 2025 and subsequently achieved major molecular response (MMR). To our knowledge, this represents the first reported case of CML harboring both a *SPECC1L* exon 4 insertion within the e8a2 transcript and a concurrent *ABL1* V379I mutation. This unique genetic profile may confer a synergistic mechanism of TKI resistance and aggressive disease behavior.

## Case presentation

A 22-year-old woman underwent treatment at an external hospital in October 2016. Lab tests showed a white blood cell count of 226.72×10^9^/L, platelet count of 107×10^9^/L, and hemoglobin level of 5.8 g/dL. Bone marrow morphological examination was consistent with CML. Cytogenetic analysis confirmed the presence of the Philadelphia chromosome, with a karyotype of 46,XX,t(9;22)(q34;q11.2). She received imatinib 400 mg daily for 3 years but discontinued it due to persistent nausea and vomiting. On 2 March 2024, she was admitted to our hospital with a ten-day history of fever, cough, and fatigue. C-reactive protein (73.90 mg/L; normal rang: 0–10 mg/L) and procalcitonin (11.39 ng/mL; normal rang: 0-0.05 ng/mL) were elevated. Abdominal ultrasound revealed hepatosplenomegaly: the left hepatic lobe measured 92 × 74mm, the right hepatic subcostal oblique diameter was 155mm, and the spleen was 208mm in length with an 84-mm hilar depth. Chest CT revealed bilateral pulmonary infiltrates indicative of pneumonia. The T-SPOT.TB assay was positive. Bronchoscopy revealed chronic inflammation in bilateral bronchi. Microbiological NGS analysis of bronchoalveolar lavage fluid identified *Enterococcus faecalis* (189 reads), *Epstein-Barr virus* (19,344 reads), and *Mycobacterium tuberculosis* (760 reads). The patient reported a prior tuberculosis (TB) exposure history, which further supports the diagnosis of secondary pulmonary tuberculosis.

The complete blood count revealed white blood cell count of 119.28×10^9^/L, platelet count of 82×10^9^/L, hemoglobin level of 3.5g/dL, granulocyte count of 85.11×10^9^/L, and monocyte count of 22.47×10^9^/L. Peripheral blood smear: blasts 14.5%, promyelocytes 1.5%, metamyelocytes 20.5%, myelocytes 13.5%, neutrophilic band forms 23.5%, segmented neutrophils 17.5%, eosinophils 2%, basophils 4%, lymphocytes 3%, intermediate normoblasts 4%, and late normoblasts 16%. Morphological analysis of bone marrow revealed markedly increased proliferation of nucleated cells, with granulocytes accounting for 87.5%, including 12.0% blast cells. Flow cytometry immunophenotyping revealed that immature cells constituted 8.68%, co-expressing CD117, CD34, CD13, HLA-DR, and MPO, with partial expression of CD33, CD38, and CD9. These results are consistent with a diagnosis of accelerated-phase CML.

**Figure 1:**
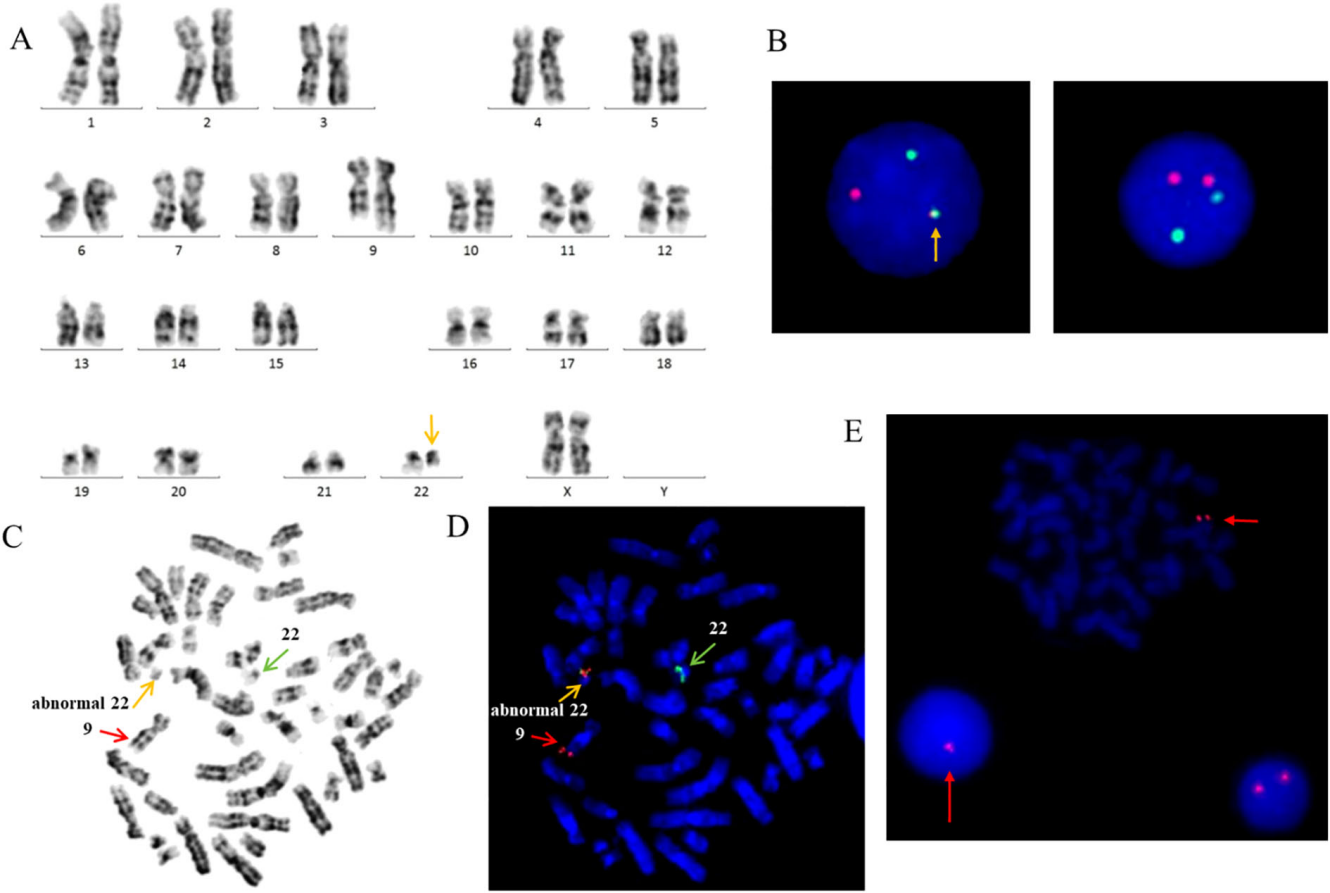
**(A)** Karyotype showing the typical translocation between chromosome 9 and 22. **(B)** FISH analysis using ABL(red) and BCR(green) dual-fusion probes. **(C)** Giemsa-banded metaphase preparation. **(D)** FISH analysis using BCR/ABL dual-fusion probe, demonstrating *BCR::ABL1* fusion signals on chromosomes 22. **(E)** FISH analysis using a LSP probe for *ASS* at 9q34 reveals a single red signal, indicative of a deletion at the ASS locus.

The typical e13a2/e14a2 transcripts, as well as the atypical e19a2 and e1a2 transcripts, were not detected by RT-PCR. However, karyotype analysis showed 46,XX,t(9;22)(q34;q11.2) in all analyzed metaphase cells (**Figure 1A**). Interphase FISH with the BCR/ABL DF probe revealed a variant 1R1G1F pattern (**Figure 1B**). Therefore, metaphase FISH was performed. The results showed 46,XX,t(9;22)(q34;q11.2)[20].ish t(9;22)(ABL1-,BCR-;BCR+,ABL1+)[20], indicating that the fusion signal was located on chromosome 22, with no corresponding signal detected on der(9) (**Figures 1C, D**). Additionally, an abnormal 1R pattern was detected using the ASS probe (**Figure 1E**).

Whole-transcriptome sequencing revealed the presence of fusion genes *SPECC1L::ABL1* (*SPECC1L* exon 4 fused to *ABL1* exon 2), *BCR::SPECC1L* (*BCR* exon 8 fused to *SPECC1L* exon 4), and *LILRB1::LILRA2*, along with a missense mutation (c.1135G > A, p.V379I) in the *ABL1* gene. The rare e8a2 *BCR::ABL1* variant was confirmed through the use of e8a2 RT-PCR primers (**Supplementary Figure 1A**). Sequence analysis of the amplified RT-PCR products demonstrated that a 154-bp sequence corresponding to exon 4 of the *SPECC1L* gene had been inserted at the e8a2 fusion junction (**Supplementary Figure 1B**).

Following six months of treatment, an Optical Genome Mapping (OGM) test was performed, the result was: ogm[GRCh38] 9q34.11q34.13(129791854_132041726)×1[0.078], t(9;22)(q34.11;q11.23)(129792636;24315572)[VAF0.18],22q11.23(23577634_24380537)×1[0.146],t(22;9)(q11.23;q34.12)(23261125;130777258)(BCR::ABL1)[VAF0.15]. The findings indicate an unbalanced translocation between chromosomes 9 and 22 involving the *BCR::ABL1* fusion gene. The variant allele frequency of this *BCR::ABL1* fusion allele was approximately 15%. Deletions of 2.250 Mb (including *ASS1*) and 0.803 Mb (including *CABIN1*) were detected at the breakpoint regions on chromosomes 22 and 9, respectively (**Supplementary Figure 2**). It is important to note that the accurate determination of these translocation breakpoints is challenging due to the inherent resolution limitations of OGM and the potential difficulty in precisely identifying unlabeled DLS regions.

After one year of treatment, whole exome sequencing analysis was performed to determine whether new clonal evolution had occurred in the patient. No additional mutations were identified in *ABL1*; however, a germline missense mutation, c.185A>G (p.His62Arg), was detected in *G6PD*. CNV-seq revealed the following copy number variations: seq[GRCh37] 9q34.11q34.12(132562879_133643956)×1,22q11.23(23597083_24677393)×1. These findings indicate a segmental deletion on chromosome 9 with the breakpoint located within the *ABL1* gene, as well as a segmental deletion on chromosome 22 involving breakpoints within the *BCR* gene (22q11.23) and the *SPECC1L* gene (22q11.23), respectively.

According to the results of various molecular biological analyses, the patient was found to have two breaks in the long arms of chromosomes 9 and 22, with sequence deletions, leading to the formation of an unbalanced translocation [t(9;22)]. Additionally, an e8a2 *BCR::ABL1* fusion transcript was detected; notably, the insertion of a 154 bp sequence from exon 4 of the *SPECC1L* gene at the fusion junction resulted in a novel in-frame variant of this transcript, designated as e8-[ins]-a2. Moreover, a p.V379I missense mutation was identified in exon 7 of the *ABL1* gene.

The patient was diagnosed with CML in the accelerated phase (AP), complicated by concurrent pneumonia. Hydroxyurea (1g twice daily) was administered to control leukocytosis, along with antimicrobial therapy and supportive blood transfusions. The patient was also diagnosed with secondary pulmonary tuberculosis and commenced on anti-tuberculosis treatment, which was discontinued in October 2024. The patient started dasatinib 140 mg once daily on March 5, 2024. After six months of treatment, the *SPECC1L::ABL1/ABL1* fusion gene ratio was 23.49% determined by Quantitative Real-time PCR, indicating resistance to dasatinib. A third-generation TKI was recommended; however, the patient declined the treatment due to financial constraints.

**Figure 2:**
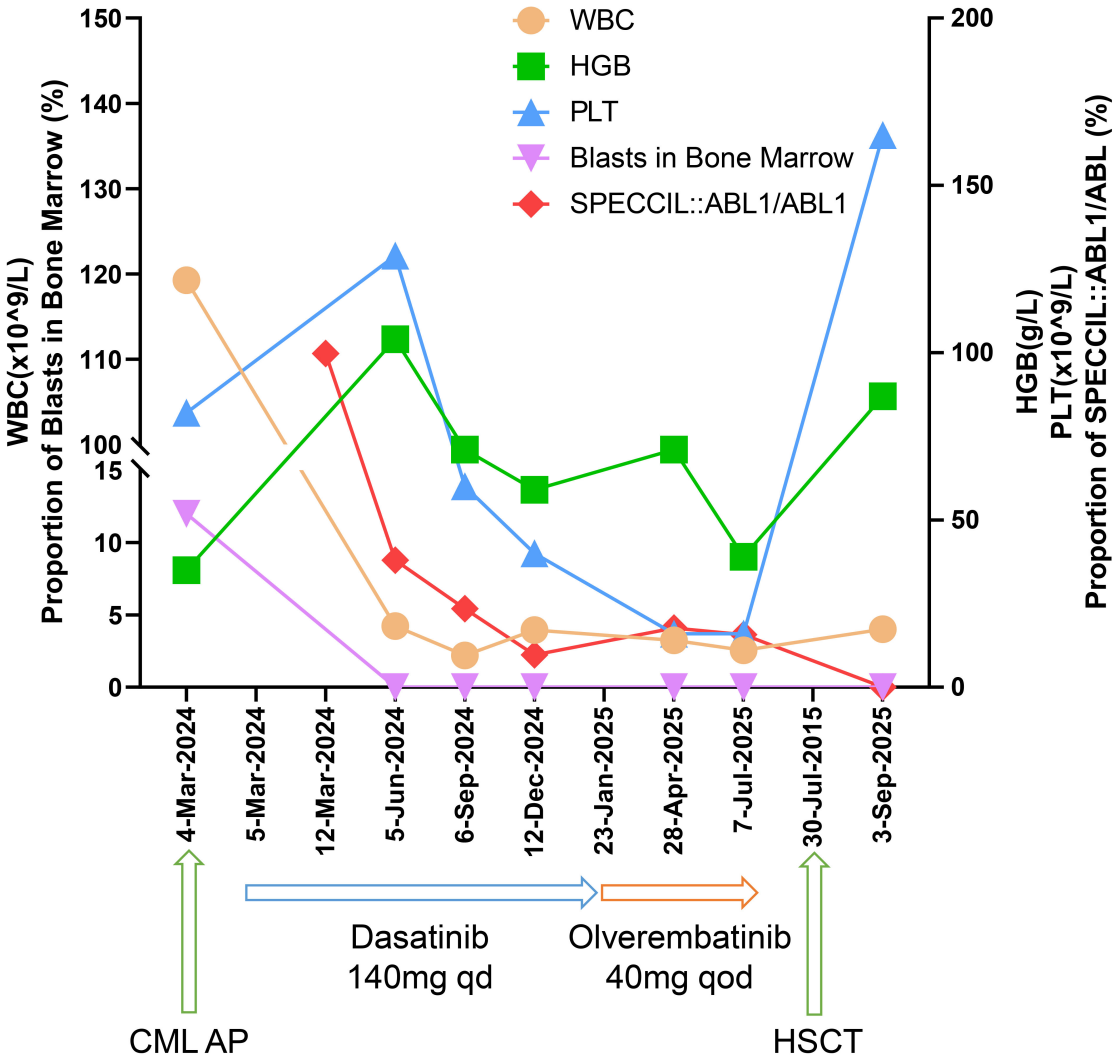
The graph illustrates the quantitative changes in leukocyte count, hemoglobin levels, platelet count, bone marrow blast percentage, and *SPECCIL::ABL1/ABL1* fusion ratio following TKI treatment.

Following nine months of continued dasatinib treatment, the fusion gene ratio decreased to 9.64%. On January 23, 2025, the patient agreed to initiate treatment with olverembatinib. After six months of olverembatinib therapy, the *SPECC1L::ABL1/ABL1* fusion gene ratios were recorded as 15.75%, indicating resistance to olverembatinib as well. The patient has undergone haplo-HSCT and has achieved a MMR. The regular monitoring results are presented in **Table 1**, and the treatment process and outcomes are summarized in **Figure 2**.

**Table 1:** Genetic and molecular characteristics of patient.

Count	Testing date	Karyotype analysis	FISH BCR/ABL and ASS detection	*SPECC1L::ABL1*/*ABL1* quantitative analysis*	Detection of *ABL1* mutations
1	2024.03.04	46,XX,t(9;22)(q34;q11.2)[20]	1R1G1F 90% 1R 92%	99.75%	*ABL1*(NM_005157) c.1135G>A (p.Val379Ile)
2	2024.06.05	46,XX,t(9;22)(q34;q11.2)[4]/46,XX[1]	1R1G1F 56% NA	37.96%	*ABL1*(NM_005157) c.1135G>A (p.Val379Ile)
3	2024.09.06	46,XX,t(9;22)(q34;q11.2)[20]	1R1G1F 37% 1R 35%	23.49%	*ABL1*(NM_005157) c.1135G>A (p.Val379Ile)
4	2024.12.12	46,XX,t(9;22)(q34;q11.2)[20]	1R1G1F 31% NA	9.64%	No mutations
5	2025.04.28	46,XX,t(9;22)(q34;q11.2)[20]	1R1G1F 38% NA	17.66%	No mutations
6	2025.07.07	NA	NA	15.75%	No mutations
7	2025.09.03	NA	NA	0.02%	NA

*Quantitative analysis was performed by Quantitative Real-time PCR.

NA, No detection; R, Red; G, Green; F, Fusion.

## Discussion

This study reports an unusual case of CML identified via whole-transcriptome sequencing. The case was defined by two concurrent genetic aberrations: a rare e8a2 *BCR::ABL1* fusion transcript harboring an in-frame insertion of *SPECC1L* exon 4, and a missense mutation (V379I) in the *ABL1* kinase domain. Neither of these molecular features is common individually, and their co-occurrence has not been previously documented in TKI-treated CML. This finding provides critical insights into multilayered mechanisms underlying therapeutic resistance in CML.

CML with e8a2 *BCR::ABL1* transcripts is relatively rare, with about 20 cases reported to date (**Table 2**). It is widely acknowledged that a direct junction between *BCR* exon 8 (e8) and *ABL1* exon 2 (a2) generates a premature stop codon (UAG) at position 7 after the fusion (5, 7). However, insertion of certain sequences can result in the formation of an in-frame e8a2 transcript. These insertion sequences generally derived from *ABL* intron Ia/Ib, *BCR* intron 8, and other specific genes including *PRDM12*, *SPECC1L*, and *MAST2*. Although these inserted sequences do not participate in encoding functional protein domains, they can indirectly affect the expression level of the BCR::ABL1 fusion protein by adjusting the splice sites of the fusion gene or regulating mRNA stability, thereby leading to differences in the clinical phenotype of CML patients and their responses to drug treatment.

**Table 2:** Summary of CML cases with e8a2 *BCR::ABL1* fusion transcript.

Patient	Age/Sex	Insertion fragment between *BCR* exon e8 and *ABL1* exon a2	Therapeutic strategy	Best response	MMR	Reference
Case1	51/M	No Insertion fragment	Hu/α-IFN	CHR	Alive(97+)	(15)
Case2	55/M	55 bp of inverted *ABL1* intron 1b	α-IFN	NA	Death(29)	(16)
Case3	56/F	31bp of *ABL1* intron 1b	α-IFN	CHR	Alive(51+)	(17)
Case4	69/F	55 bp of inverted *ABL1* intron 1b	Hu	PHR	Alive(48+)	(18)
Case5	56/M	55 bp of inverted *ABL1* intron 1b	Busulfan/Hu	CHR	Death(89)	(5)
Case6	75/F	151 bp of *ABL1* intron 1a	IM/cytarabine	CHR	Alive(4+)	(5)
Case7	46/F	91 bp from *KIAA0376*(*SPECC1l*) gene	Hu/α-IFN	PHR	Alive(5+)	(5)
Case8	40/M	55 bp of inverted *ABL1* intron 1b	IM	CCyR and MMR	MMR(33)	(19)
Case9	35/M	55 bp of inverted *ABL1* intron 1b	IM	CCyR and MMR	MMR(30)	(19)
Case10	47/M	55 bp of inverted *ABL1* intron 1b	IM	CCyR and MMR	MMR(39)	(19)
Case11	43/M	46 bp of *BCR* intron 8	IM	CCyR and MMR	MMR(12)	(20)
Case12	46/M	14 bp of *ABL1* intron 1a	IM	CHR	CHR(3)	(21)
Case13	40/F	16 bp of *ABL1* intron 1a	α-IFN/IM	CHR and PMR	PMR(65)	(22)
Case14	51/F	148 bp from *MAST2* gene	IM	CCyR and MMR	MMR(24)	(23)
Case15	67/M	112 bp from *PRDMI2* gene	NI/IM	CMR	CMR(82)	(3)
Case16	58/F	154 bp from *SPECC1L* gene	IM	CCyR and MMR	MMR(66)	(3)
Case17	25/F	27 bp of *BCR* intron 8	Hu/DA	CCyR and EMR	EMR(3+)	(24)
Case18	47/F	No Insertion fragment	IM	CMR	CMR(17)	(6)
Case19	74/M	55 bp of inverted *ABL1* intron 1b	Hu/NI	MMR	MMR(12)	(7)
Case20	45/F	55 bp of inverted *ABL1* intron 1b	FM/Hu	MMR	MMR(17)	(25)

α-IFN, interferon-alpha; CCyR, complete cytogenetic response; CHR, complete hematological response; CMR, complete molecular response; DA, dasatinib; EMR, early molecular response; F, female; FM, flumatinib; Hu, hydroxyurea; IM, imatinib; M, male; MMR, major molecular; NI, nilotinib; NA, not available; PHR, partial hematological response; PMR, partial molecular response.

Among these, the insertion of *SPECC1L* sequences into the e8a2 transcript is rare, with only two documented cases reported prior to this study (3, 5). In these two earlier studies, the patients primarily received treatment with interferon-α or imatinib, achieving only partial hematological response and MMR, respectively. Our case represents the first documented instance of this specific genotype being sequentially treated with second- and third-generation TKIs (dasatinib and olverembatinib). Notably, the patient also harbored an *ABL1* V379I mutation, allowing for a comparative assessment of therapeutic efficacy against this genetic background.

The poor treatment response observed in our patient is likely driven primarily by the genetic alterations. The V379I mutation is located between the catalytic domain and the activation loop of the ABL1 kinase, where it plays a critical role in abrogating binding to BCR::ABL1 (8). To date, this mutation has been reported in only two cases (8, 9). One patient was treated with imatinib for 17 years, achieving a complete hematological response but failing attain a MMR. The other patient progressed to the accelerated phase after 34 months of imatinib treatment, at which point the V379I mutation was identified. Treatment was switched to dasatinib; 10 months later, a compound mutation involving V379I and T315I emerged. Like the second reported case, our patient progressed to the accelerated phase following imatinib treatment and later harbored the V379I mutation. Subsequently, treatment with dasatinib and olverembatinib failed to achieve disease control. In conjunction with previously reported findings, this observation suggests that the V379I mutation may confer resistance not only to first- and second-generation TKIs but also to the third-generation agent olverembatinib. Furthermore, the insertion of *SPECC1L* may contribute to increased resistance to drug therapy. *SPECC1L* is critical for cytoskeletal organization, cytokinesis, and cell migration (3, 10). Aberrant integration of *SPECC1L* may contribute to genomic instability and could potentially impair TKI binding through an allosteric effect. Most critically, we propose that the co-occurrence of the V379I mutation and the *SPECC1L* insertion engenders a synergistic resistance phenotype. Neither alteration alone may be sufficient to confer broad TKI resistance, but together they likely destabilize the ABL1 kinase domain in a manner that precludes effective drug binding. This model is strongly corroborated by the failure of olverembatinib, despite its documented activity against most single ABL1 mutations (11), to control the disease in our patient.

Certain clinical factors may also influence the treatment outcomes of patients. The advanced phase of disease (AP) at the time of second-line treatment, the prior history of TKIs exposures, and the prolonged interval from diagnosis to the initiation of olverembatinib are all associated with a diminished therapeutic response. Furthermore, a drug-drug interaction with rifampicin may have initially reduced dasatinib exposure, but the persistence of resistance after the discontinuation of rifampicin confirms an inherent resistance mechanism (12).

It is important to emphasize that olverembatinib demonstrates notable efficacy and favorable safety in the treatment of CML. It exhibits anti-leukemic activity against nearly all ABL1 kinase domain mutations that cause TKI resistance (11). However, the therapeutic efficacy in patients in the accelerated phase is lower than that in patients in the chronic phase (13). The treatment failure in this case highlights the potential clinical severity of synergism between a *SPECC1L* insertion and the V379I mutation in the kinase domain, which may compromise the efficacy of even third-generation TKI.

Finally, among patients with CML, about 10-15% have been reported to have deletions adjacent to the t(9;22) breakpoint on der(9). CML-AP patients who carry this deletion may demonstrate a suboptimal response to imatinib. This may be attributed to the loss of critical genes, such as tumor suppressor genes, within the deleted region and is associated with increased genomic instability (14).

In conclusion, this case underscores the clinical imperative of utilizing molecular diagnostics, particularly whole-transcriptome sequencing, in instances where conventional *BCR::ABL1* assays yield negative results despite cytogenetic evidence of Ph+ leukemia. For patients with atypical e8a2 transcript, particularly younger individuals, the earlier administration of second- or third-generation TKIs, in conjunction with *ABL1* kinase domain mutation analysis, may yield more favorable clinical outcomes. Larger, multicenter studies are necessary to establish the most effective treatment strategy for patients with these rare transcripts.

## Data Availability

The original contributions presented in the study are included in the article/**Supplementary Material**. Further inquiries can be directed to the corresponding authors.
