# Supplementary material for: Case Report: Dual resistance to dasatinib/olverembatinib in accelerated-phase cml: identification of a novel SPECC1L-inserted e8a2 BCR::ABL1 transcript and ABL1 V379I mutation
Source: Front Oncol. 2025 Oct 24;15:1711888. doi: 10.3389/fonc.2025.1711888 (PMC12591956; doi:10.3389/fonc.2025.1711888)
Supplement: Supplementary file 1 [file DataSheet1.docx]

**Figures**


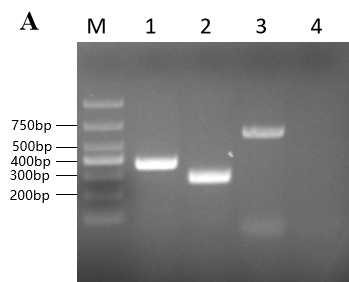

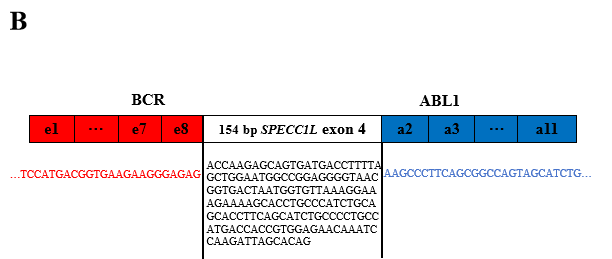


Figure 1 Molecular analysis of the patient. (A) EB-stained 2% agarose gel of RT-PCR for this e8a2 *BCR::ABL1* transcript. Lane M, PCR Marker; Lane 1, e13a2 *BCR::ABL1* positive control; Lane 2, Detection with *SPECC1L/ABL1* primers (248 bp); Lane 3, Detection with e8a2 primers (617 bp); Lane 4, Water negative control. (B) Sequence analysis of the whole-transcriptome identified the same e8a2 *BCR::ABL1* transcript, which contains an insertion of *SPECC1L* exon 4 (accession number NM_015330.6) at the fusion junction.

**A**


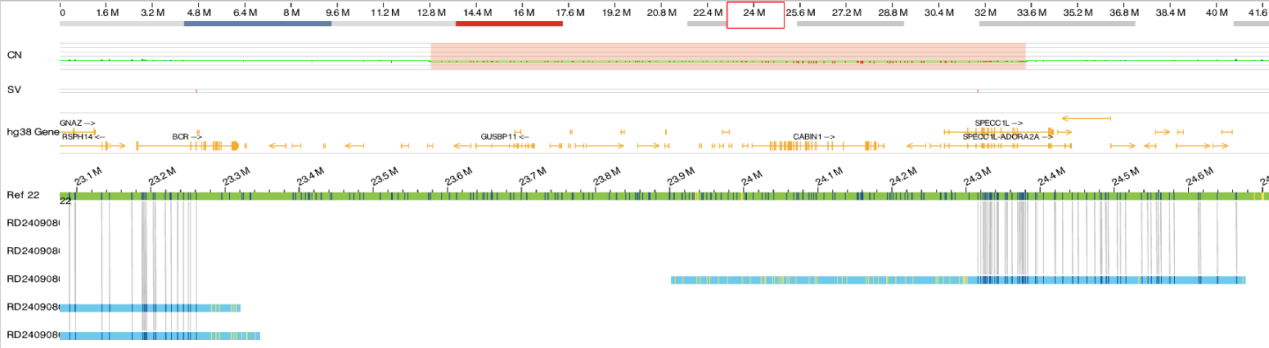


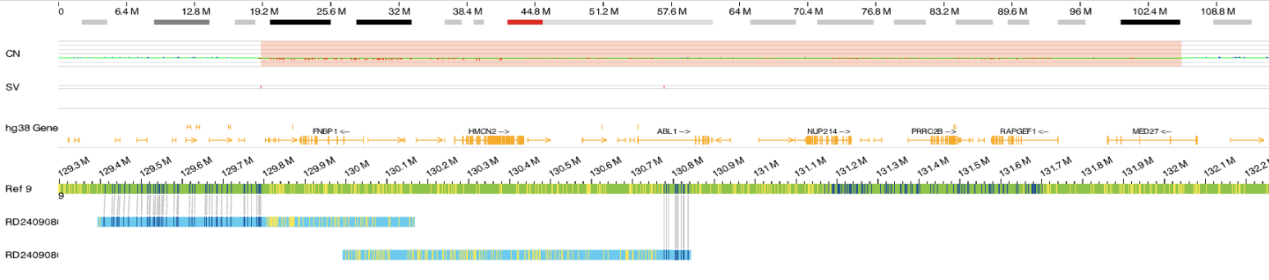


**B**


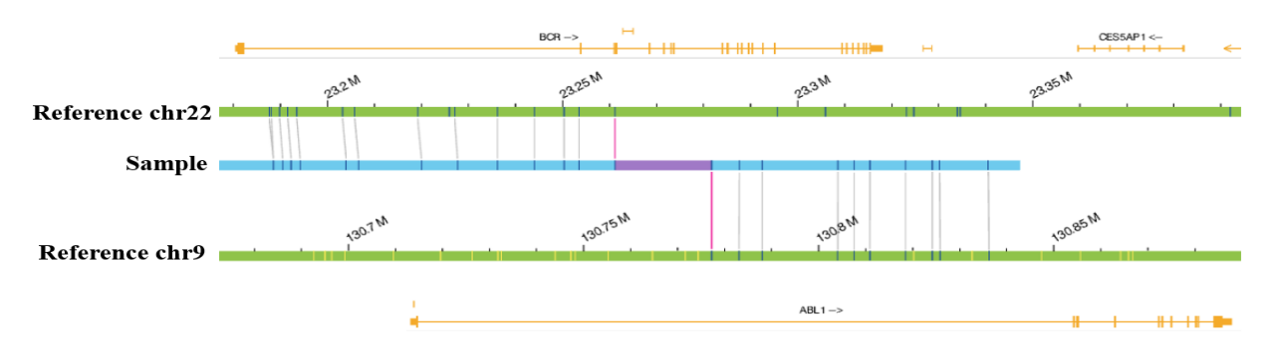


**D**

**C**


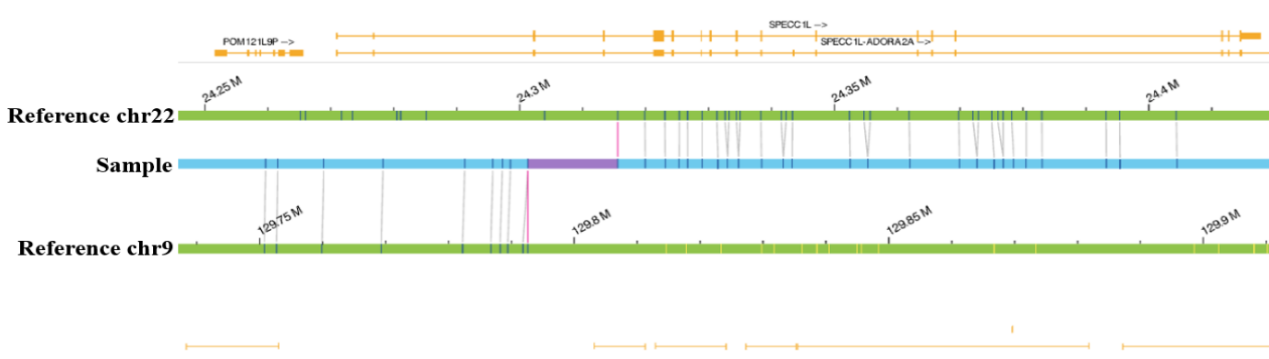


Figure 2 The OGM analysis of the patient. (A) The long arm of chromosome 22 exhibits two distinct locations: q11.23 (23.261 Mb) and q11.23 (24.316 Mb). (B) The long arm of chromosome 9 exhibits two distinct locations: q34.11 (129.793 Mb) and q34.12 (130.777 Mb). (C) The breakpoint at 22q11.23 (23.261 Mb) (and its upstream region) and the breakpoint at 9q34.12 (and its downstream region) have undergone translocation and recombination, resulting in a *BCR::ABL1* fusion gene with an undefined sequence in the intervening segment. (D) Translocation and recombination events involving 9q34.11 (and upstream regions) and 22q11.23 (24.316 Mb) (and downstream segments) did not produce a fusion gene.
